# Clinical significances of hsa_circ_0067582 and hsa_circ_0005758 in gastric cancer tissues

**DOI:** 10.1002/jcla.22984

**Published:** 2019-07-22

**Authors:** Rongdan Lu, Yongfu Shao, Xueping Tao, Guoliang Ye, Bingxiu Xiao, Junming Guo

**Affiliations:** ^1^ Department of Gastroenterology The Affiliated Hospital of Medical School of Ningbo University Ningbo China; ^2^ Department of Biochemistry and Molecular Biology and Zhejiang Key Laboratory of Pathophysiology Ningbo University School of Medicine Ningbo China

**Keywords:** circular RNA, clinical significance, gastric cancer, Hsa_circ_0005758, Hsa_circ_0067582

## Abstract

**Background:**

Circular RNAs (circRNAs) are a special class of endogenous noncoding RNAs that have numerous biological functions in normal situation and diseases including cancers. However, the clinical significance of circRNAs in gastric cancer (GC) remains largely unknown. Here, we chose two representative circRNAs, hsa_circ_0067582 and hsa_circ_0005758, to investigate their clinical significance in GC patients.

**Methods:**

Using real‐time quantitative reverse transcription‐polymerase chain reaction (qRT‐PCR), we explored the expression levels of hsa_circ_0067582 and hsa_circ_0005758 in tissues with different stages of gastric tumorigenesis. Then, the relationships between their expression levels and GC patients' clinicopathological factors were further investigated. Receiver operating characteristic (ROC) curves were established for evaluating diagnostic values of hsa_circ_0067582 and hsa_circ_0005758.

**Results:**

Compared with healthy control tissues, both hsa_circ_0067582 and hsa_circ_0005758 were significantly decreased in GC tissues. Besides, hsa_circ_0067582 expression was associated with GC patients' tissue CEA level (*P* <.001) and stages (*P* = .037); and hsa_circ_0005758 expression was relevant to tissue CEA level (*P* < .001) and perineural invasion (*P* = .048). The area under the ROC curve (AUC) of hsa_circ_0067582 was up to 0.671. The cutoff value was set at 10.61, with which the sensitivity and specificity were 55.2% and 75.0%, respectively. Similar to hsa_circ_0005758, the AUC of hsa_circ_0005758 was 0.721. The cutoff value was set at 10.20, with which the sensitivity and specificity were 75.0% and 67.7%, respectively.

**Conclusion:**

These results showed that both hsa_circ_0067582 and hsa_circ_0005758 may play an important role in gastric carcinogenesis; and they may be potential indicators for GC diagnosis.

## 1 INTRODUCTION

Gastric cancer (GC) is one of the most common malignancies and the second leading cause of cancer death in the world.^1^ Mortality rates are higher in Asian countries, and patients are usually diagnosed at later stages, leading to a very low survival rate for the less understanding of cancer heterogeneity and absence of desirable biomarkers for early detection.^2^, ^3^ Nowadays, the main treatment of gastric cancer is radical surgical resection, while some patients also need chemotherapy, radiotherapy, and other treatment modes. Despite those therapeutic options available, overall prognosis of gastric cancer remains poor.^4^ Therefore, to better understand the potential molecular mechanism of carcinogenesis and development of gastric cancer and find new diagnostic targets for clinical screening are of great significance.^5^

Circular RNAs (circRNAs) are a special class of endogenous noncoding RNAs that have numerous biological functions with their features of conservation, stability, abundance, and tissue‐specific expression in organisms.^2^ Some circRNAs might regulate microRNA (miRNA) function as microRNA sponges and the circRNA‐miRNA‐mRNA axis may play an indirect role in the regulation on post‐transcriptional level.^6^ Many studies showed that circRNAs play important roles in human diseases, such as circ‐Foxo3 in cardiac senescence, ciR‐7 in Alzheimer's disease, and cir‐ITCH in colorectal cancer.^7^ CircRNAs exert distinct effects, both as tumor suppressors and oncogenes.^6^ CircRNAs appear to be more often down‐regulated in tumor tissues compared to normal tissues.^8^ In our previous study, we analyzed the gene expression profiles of GC and paired normal tissue samples, and identified a number of genes that are significantly up‐regulated or down‐regulated in cancer tissues compared with their adjacent normal tissues.^2^

Hsa_circ_0067582 and hsa_circ_0005758 are gastric cancer‐associated circRNAs based on bioinformatics analysis. Hsa_circ_0067582, 394 nt in length, is transcripted from chr3:141231004‐141259451. Its associated gene symbol is RASA2 (RAS p21 protein activator 2). Hsa_circ_0005758 is a circRNA with 373 nt in length. Its gene is located at chr1:155891165‐155893478, with associated gene symbol KIAA0907. They are both among the most deregulated circRNAs in gastric cancer.

In this study, the expression of hsa_circ_0067582 and hsa_circ_0005758 in GC tissue specimens and paired normal tissues were first explored. Then, their expression levels in tissues with different stages of gastric tumorigenesis were measured. The potential relationships between circRNAs' expression levels and patients' clinicopathological factors were further investigated to gain a better understanding of their biological roles in gastric cancer. Finally, ROC curves were established for evaluating their diagnostic values. Our results indicated that both hsa_circ_0067582 and hsa_circ_0005758 may play an important role in gastric carcinogenesis, and they may be potential indicators for GC diagnosis.

## 2 MATERIALS AND METHODS

### 2.1 Specimens and clinical data collection

A total of 263 samples were collected from the Affiliated Hospital of Medical School of Ningbo University, China, from November 2012 to June 2016. All of the patients underwent neither chemotherapy nor radiotherapy before operation. The 96 GC tissues and their adjacent non‐tumorous tissues 5 cm from the edge of the tumor were obtained from surgical operations. Tumors were staged according to the tumor‐node‐metastasis (TNM) stage system of the international Union Against Cancer (7th ed). Histological grade was assessed following the National Comprehensive Cancer Network (NCCN) clinical practice guideline of oncology (V.1.2011). Another 29 human healthy gastric mucosa, 29 gastritis mucosa, and 13 gastric intestinal metaplasia tissues were gained from biopsy specimens. All samples were quickly frozen in RNA‐fixer Reagent (Betake) and stored at −80°C until further experiments. Each specimen was histopathologically confirmed. All human studies were approved by the Institute Research Medical Ethics Committee of the Affiliated Hospital of Medical School of Ningbo University. Written informed consent was obtained from all subjects.

### 2.2 Total RNA preparation and qRT‐PCR detection

Total RNA was extracted from specimens by TRIzol reagent (Ambion), then reverse transcribed into cDNA by GoScript Reverse Transcription (RT) System (Promega) following the manufacturer's instructions. Real‐time quantitative reverse transcription‐polymerase chain reaction (qRT‐PCR) was used to measure the expression levels of has_circ_0067582 and has_circ_0005758 in all samples by using GoTaq qPCR Master Mix (Promega) on an Mx3005P real‐time PCR System (Stratagene), according to the manufacturer's protocol. Primers for hsa_circ_0067582, hsa_circ_0005758 and glyceraldehyde 3‐phosphate dehydrogenase (GAPDH) were synthesized by Sangon Biotech. The sequences of the PCR primers were as follows: 5′‐CACAGTGGCAAAGAAACTTGGT‐3′ and 5′‐TGTGGGGTCCAAGATATGGC‐3′ for hsa_circ_0067582; 5′‐CCCACCTGTGCCAACACAAT‐3′ and 5′‐CAGCCAGGCCTTCTGGTTTG‐3′ for hsa_circ_0005758; 5′‐ACCCACTCCTCCACCTTTGAC‐3′ and 5′‐TGTTGCTGTAGCCAAATTCGTT‐3′ for GAPDH, as normalize control. The conditions of thermal cycling were as follows: 95°C at 5 miutes for a hot‐start, then 40 cycles at 94°C for 15 seconds, 55°C for 30 seconds, and 72°C for 30 seconds. The cycle threshold (*C*_t_) values were recorded for hsa_circ_0067582, hsa_circ_0005758, and GAPDH. The data were analyzed through the Δ*C*_t_ method. All results were expressed as mean ± SD of three independent experiments. Larger Δ*C*_t_ value indicates lower expression. The PCR products of both hsa_circ_0067582 and hsa_circ_0005758 were confirmed by sequencing (Figure S1).

### 2.3 Immunohistochemical analysis of tissue CEA and CA19‐9

We incubated the paraffin tissue sections in primary anti‐carcinoembryonic antigen (CEA) or anti‐carbohydrate antigen 19‐9 (CA19‐9) (DAKO) for 1 hour at room temperature; then we incubated the tissues in diaminobenzidine (DAB; DAKO) for color development after incubation with broad spectrum second antibody K5007 (DAKO). The results were classified as negative or positive (Figure S2).

### 2.4 Statistical analysis

All statistical analyses were performed by Statistical Program for Social Sciences 20.0 software (SPSS), GraphPad Prism 5.0 (GraphPad Software), and SigmaPlot 10.0 (SigmaPlot Software). We used Student's *t* test, one‐way analysis of variance (ANOVA) test, and rank‐sum test as appropriate. Statistical significance was accepted at *P* < .05.

## 3 RESULTS

### 3.1 Hsa_circ_0067582 and hsa_circ_0005758 were down‐regulated in GC tissues

**Figure 1:**
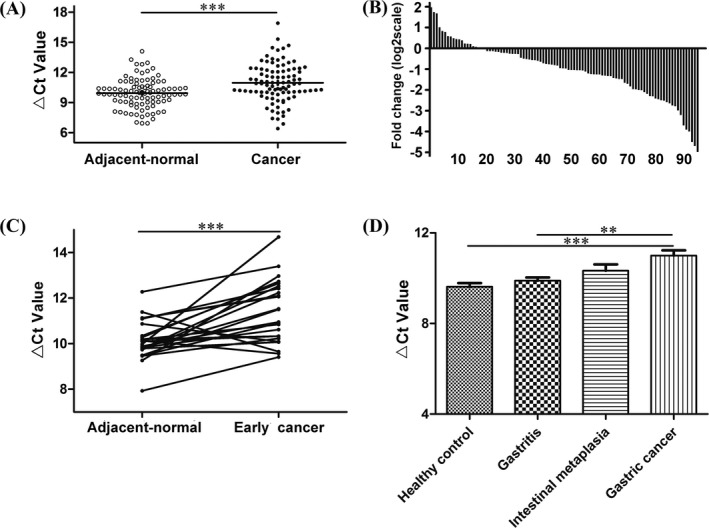
Hsa_circ_0067582 expression levels in gastric cancer tissues. A, The expression levels of hsa_circ_0067582 in cancer tissues (n = 96) and adjacent normal tissues (n = 96). B, The expression level of hsa_circ_0067582 was significantly down‐regulated in 80.2% (77/96) gastric cancer tissues compared with the adjacent normal tissues. C, The hsa_circ_0067582 expression levels in early gastric cancer (n = 24) compared with the adjacent normal (n = 24) tissues. D, The hsa_circ_0067582 expression levels in healthy gastric mucosa (n = 29), gastritis mucosa (n = 29), gastric intestinal metaplasia (n = 13), and gastric cancer (n = 96) tissues were determined by qRT‐PCR. Higher ^Δ^ *C*t value indicates lower expression (*P < .05, **P < .01, ***P < .001)

**Figure 2:**
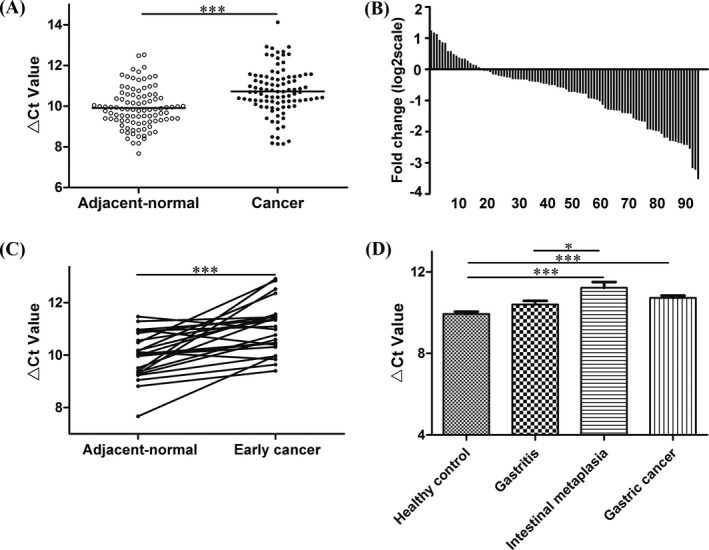
Hsa_circ_0005758 expression levels in gastric cancer tissues. A, The expression levels of hsa_circ_0005758 in cancer tissues (n = 96) and adjacent normal tissues (n = 96). B, The expression level of hsa_circ_0005758 was significantly down‐regulated in 81.3% (78/96) gastric cancer tissues compared with the adjacent normal tissues. C, The hsa_circ_0005758 expression levels in early gastric cancer (n = 24) compared with the adjacent normal (n = 24) tissues. D, The hsa_circ_0005758 expression levels in healthy gastric mucosa (n = 29), gastritis mucosa (n = 29), gastric intestinal metaplasia (n = 13), and gastric cancer (n = 96) tissues were determined by qRT‐PCR. Higher Δ*C*t value indicates lower expression (*P < .05, **P < .01, ***P < .001)

After the verification by qRT‐PCR method, hsa_circ_0067582 and hsa_circ_0005758 were both found markedly down‐regulated in GC tissues compared with the adjacent non‐tumorous tissues (Figure 1A, Figure 2A). Hsa_circ_0067582 and hsa_circ_0005758 expression levels were significantly down‐regulated in 80.2% (77/96) and 81.3% (78/96) GC tissues, respectively. (Figure 1B, Figure 2B). Furthermore, the expression of them were both lower in the early GC tissues than the matched adjacent tissues (Figure 1C, Figure 2C). Compared with healthy control group, hsa_circ_0067582 and hsa_circ_0005758 expression levels were significantly decreased in GC tissues (Figure 1D, Figure 2D). Besides, hsa_circ_0005758 expression level was remarkably decreased in GIM tissues compared with gastritis group (Figure 2D). There was no significant difference in the level of hsa_circ_0067582 between GIM group and gastritis group (Figure 1D).

### 3.2 Relationship between hsa_circ_0067582, hsa_circ_0005758 levels, and clinicopathological factors

The correlations between hsa_circ_0067582, hsa_circ_0005758 expression, and clinicopathological features of GC patients were further analyzed. The results indicated that low expression of hsa_circ_0067582 was associated with GC patients' tissue CEA level (*P* < .001) and stages (*P* = .037). Although low expression of hsa_circ_0005758 was relevant to tissue CEA level (*P* < .001) and perineural invasion (*P* = .048) (Table 1), both hsa_circ_0067582 and hsa_circ_0005758 expression level were not significantly correlated with age, gender, diameter, tissue CA19‐9 level, and so on.

**Table 1:** Relationship of CircRNAs Expression Levels (Δ*C*_t_) in Cancer Tissues with clinicopathological Factors of GC Patients

Characteristics	No.of case (%)	Hsa_circ_0067582		hsa_circ_0005758	
		Mean ± SD	*P* value	Mean ± SD	*P* value
Age (y)					
≥60	61 (63.5)	10.956 ± 2.075	.997	10.760 ± 1.213	.667
＜60	35 (36.5)	10.955 ± 1.550		10.655 ± 1.049	
Gender					
Male	65 (67.7)	10.993 ± 2.001	.785	10.668 ± 1.216	.512
Female	31 (32.3)	10.879 ± 1.667		10.834 ± 1.012	
Tumor location					
Sinuses ventriculi	49 (51.1)	10.858 ± 1.869	.592	10.661 ± 1.182	.947
Cardia	10 (10.4)	10.431 ± 1.353		10.732 ± 1.359	
Corpora ventriculi	25 (26.0)	11.113 ± 1.748		10.763 ± 0.945	
Others	12 (12.5)	11.464 ± 2.621		10.874 ± 1.362	
Diameter (cm)					
≥5	47 (49.0)	11.139 ± 1.948	.357	10.904 ± 1.273	.130
＜5	49 (51.0)	10.780 ± 1.839		10.547 ± 1.004	
Differentiation					
Well	12 (12.5)	11.457 ± 1.352	.603	10.505 ± 1.123	.781
Moderate	47 (49.0)	10.837 ± 1.725		10.738 ± 1.058	
Poor	37 (38.5)	10.944 ± 2.231		10.771 ± 1.291	
Stage					
Early	24 (25.0)	11.408 ± 1.524	.037	11.025 ± 0.981	.137
Advanced	72 (75.0)	10.405 ± 1.986		10.621 ± 1.192	
Borrmann type					
I ＆ II	19 (26.4)	10.846 ± 2.739	.934	10.763 ± 1.628	.633
III ＆ IV	53 (73.6)	10.790 ± 1.669		10.570 ± 1.006	
Pathologic diagnosis					
Signet ring cell cancer	15 (15.6)	10.494 ± 2.163	.306	10.632 ± 1.112	.744
Adenocarcinoma	81 (84.4)	11.041 ± 1.839		10.739 ± 1.165	
Invasion					
T_1_ ＆ T_2_	36 (37.5)	11.266 ± 1.862	.215	10.959 ± 1.089	.119
T_3_ ＆ T_4_	60 (62.5)	10.770 ± 1.901		10.580 ± 1.174	
Lymphatic metastasis					
N_0_	38 (39.6)	11.242 ± 1.920	.233	10.944 ± 1.097	.127
N_1‐3_	58 (60.4)	10.768 ± 1.866		10.576 ± 1.172	
Distal metastasis					
M_0_	82 (85.4)	10.938 ± 1.813	.830	10.770 ± 1.079	.326
M_1_	14 (14.6)	11.057 ± 2.380		10.441 ± 1.528	
Venous invasion					
Absent	53 (55.2)	10.874 ± 1.854	.642	10.760 ± 1.149	.719
Present	43 (44.8)	11.056 ± 1.955		10.674 ± 1.167	
Perineural invasion					
Absent	47 (49.0)	11.082 ± 1.844	.525	10.916 ± 1.095	.048
Present	49 (51.0)	10.835 ± 1.948		10.346 ± 1.184	
CEA(Tissue)					
Positive	74 (77.1)	11.435 ± 1.589	＜.001	10.935 ± 1.060	＜.001
Negative	22 (22.9)	9.345 ± 1.972		10.003 ± 1.180	
CA19‐9(Tissue)					
Positive	54 (56.3)	11.039 ± 1.627	.626	10.733 ± 1.096	.916
Negative	42 (43.7)	10.848 ± 2.202		10.708 ± 1.233	

### 3.3 ROC curve of hsa_circ_0067582 and hsa_circ_0005758

**Figure 3:**
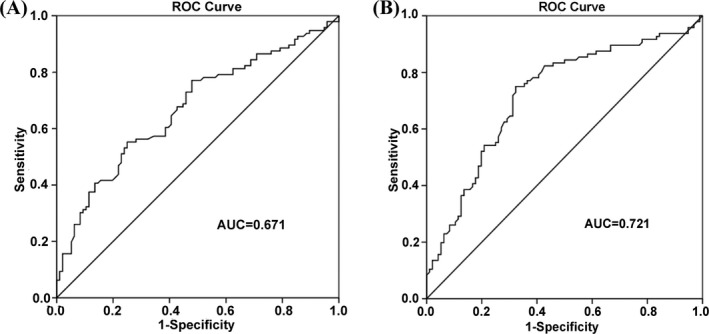
ROC curve of hsa_circ_0067582 and hsa_circ_0005758 in differentiating gastric cancer tissues from controls. A, The area under the curve (AUC) of hsa_circ_0067582 was up to 0.671. B, The AUC of hsa_circ_0005758 was up to 0.721

Receiver operating characteristic (ROC) curves were generated to evaluate the diagnostic value of circRNAs. The area under the ROC curve (AUC) of hsa_circ_0067582 was up to 0.671 (95% confidence interval [CI] = 0.595‐0.748, *P* < .0001). When the cutoff value was set at 10.61, the sensitivity and specificity of hsa_circ_0067582 were 55.2% and 75.0%, respectively (Figure 3A). Similar to hsa_circ_0067582, the AUC of hsa_circ_0005758 was 0.721 (95% confidence interval [CI] = 0.674‐0.794, *P* < .0001). When the cutoff value was set at 10.20, the sensitivity and specificity of hsa_circ_0005758 were 75.0% and 67.7%, respectively (Figure 3B).

## 4 DISCUSSION

CircRNAs, form a covalently closed continuous loop through connecting 5′ splice site to 3′ splice site, are previous regarded as functionless by‐products and have become a new hot topic in very recent years with the RNA‐seq technology of next‐generation sequencing development.^9^, ^10^, ^11^, ^12^ CircRNAs can affect the regulation of gene expression through acting as competing endogenous RNA (ceRNAs) with the characteristics of stable structure, abundance, and tissue/developmental‐stage‐specific expression.^10^, ^11^ Previous studies demonstrated that circRNAs existed widely in all kinds of organizations and exhibit abnormal expression levels in digestive system cancers.^13^ Guo et al^14^ identified that circ‐ZNF652 could induce snail‐mediated epithelial mesenchymal transition, thereby promoting hepatocellular carcinoma metastasis. Weng et al^15^ found that ciRS‐7 was a promising prognostic biomarker in colorectal cancer patients and may be served as a therapeutic target for reducing EGFR‐RAF1 activity in colorectal cancer patients. Li et al^16^ found that circMAT2B could affect the expression level of PKM2, which encodes a key enzyme in glycolytic cycle, then accelerates hepatocellular carcinoma progression, and it may provide a therapeutic target for cancer treatment. These results intensely indicate that circRNAs may play an important role in cancer for pathophysiology and clinical applications. Hsa_circ_0067582 and hsa_circ_0005758 are gastric cancer‐associated circRNAs based on our bioinformatics analysis. We first verify the expression levels of hsa_circ_0067582 and hsa_circ_0005758 in GC tissues and found that both of them were significantly down‐regulated in GC tissues compared with the paired non‐tumorous tissues (Figure 1A and 2A). Furthermore, the expression of them were also both lower in the early GC tissues than the matched adjacent tissues (Figures 1C and 2C). As the prognosis of advanced GC is poor, the 5‐year survival rate of early gastric cancer (EGC) that being performed by radical operations can be higher than 90%, even close to 100% for intramucosal invasive EGC.^17^ The research showed that these two circRNAs may be used as potential indicators for early gastric cancer.

As it is a gradual progression from inflammation to atrophic gastritis, metaplasia, dysplasia, and finally to adenocarcinoma,^18^ gastric intestinal metaplasia (GIM) is a premalignant stage in the Correa's cascade. Pittayanon et al^19^ found that incomplete GIM is an important risk factor in predicting the development of high‐grade dysplasia and/or gastric cancer. We also explored hsa_circ_0067582 and hsa_circ_0005758 expression levels in different stages of gastric carcinogenesis. Compared with healthy control group, hsa_circ_0067582 and hsa_circ_0005758 expression levels were significantly decreased in GC tissues, but there was no significant difference between healthy control group and gastritis group (Figures 1D and 2D). These results showed that low expression levels of hsa_circ_0067582 and hsa_circ_0005758 in tissues are closely related to gastric cancerogenesis.

Tumor markers, such as carcinoembryonic antigen (CEA), carbohydrate antigen 19‐9 (CA 19‐9), and carbohydrate antigen 72‐4 (CA 72‐4), which are simple and easy for screening tumors, have been widely used for the diagnosis of different types of cancers, including gastric cancer.^20^ However, these markers have low sensitivity, the sensitivity in patients with recurrences was 44% for CEA and 56% for CA 19‐9.^21^ In our study, we constructed ROC curves to evaluate the clinical diagnostic value of hsa_circ_0067582 and hsa_circ_0005758. For hsa_circ_0067582, the AUC was up to 0.671, with sensitivity and specificity 55.2% and 75.0%, respectively (Figure 3A). While for hsa_circ_0005758, the AUC was 0.721, with the sensitivity and specificity 75.0% and 67.7%, respectively (Figure 3B). The results showed that hsa_circ_0067582 and hsa_circ_0005758 were better than the traditional biomarkers of gastric cancer, and hsa_circ_0005758 had a better distinguishing value than hsa_circ_0067582.

Tumor progression is not only influenced by the reaction of the host to malignancy, but also depends on tumor clinicopathological features. A study conducted by Wang et al^22^ revealed that tissue CEA in GC is significantly correlated with preoperative serum CEA levels, depth of invasion, lymph node metastasis, distant metastasis, and TNM stages. Deng et al^23^ found that perineural invasion is an independent prognostic factor affecting overall survival of GC patients, and is independent of lymph node status, the depth of invasion and other clinicopathological features. In our study, we performed an analysis to evaluate the relationship between hsa_circ_0067582, hsa_circ_0005758 expression levels and clinicopathological factors of GC patients. Our research indicated that low expression of hsa_circ_0067582 was associated with GC patients' tissue CEA level and tumor stages, while low expression of hsa_circ_0005758 was relevant to tissue CEA level and perineural invasion (Table 1). Our data indicated that hsa_circ_0067582 and hsa_circ_0005758 may be potential potential biological molecules for clinical prognosis prediction.

Conclusively, our results suggested that hsa_circ_0067582 and hsa_circ_0005758 are closely related to gastric carcinogenesis; and the circRNAs we investigated may be potential indicators of GC.

## CONFLICT OF INTEREST

The authors declared no conflicts.

## ETHICAL APPROVAL

The study was approved by the Institute Research Medical Ethics Committee of the Affiliated Hospital of Medical School of Ningbo University.
