# Supplementary material for: Clinical significances of hsa_circ_0067582 and hsa_circ_0005758 in gastric cancer tissues
Source: J Clin Lab Anal. 2019 Jul 22;33(9):e22984. doi: 10.1002/jcla.22984 (PMC6868420; doi:10.1002/jcla.22984)

**Supplementary material**

Figure S1. The dissolution curves and product sequencing results of tissue hsa_circ_0067582 and hsa_circ_0005758. (A) The dissolution curve of tissue hsa_circ_0067582. (B) The dissolution curve of tissue hsa_circ_0005758. (C) The sequencing result of hsa_circ_0067582. (D) The sequencing result of hsa_circ_0005758.


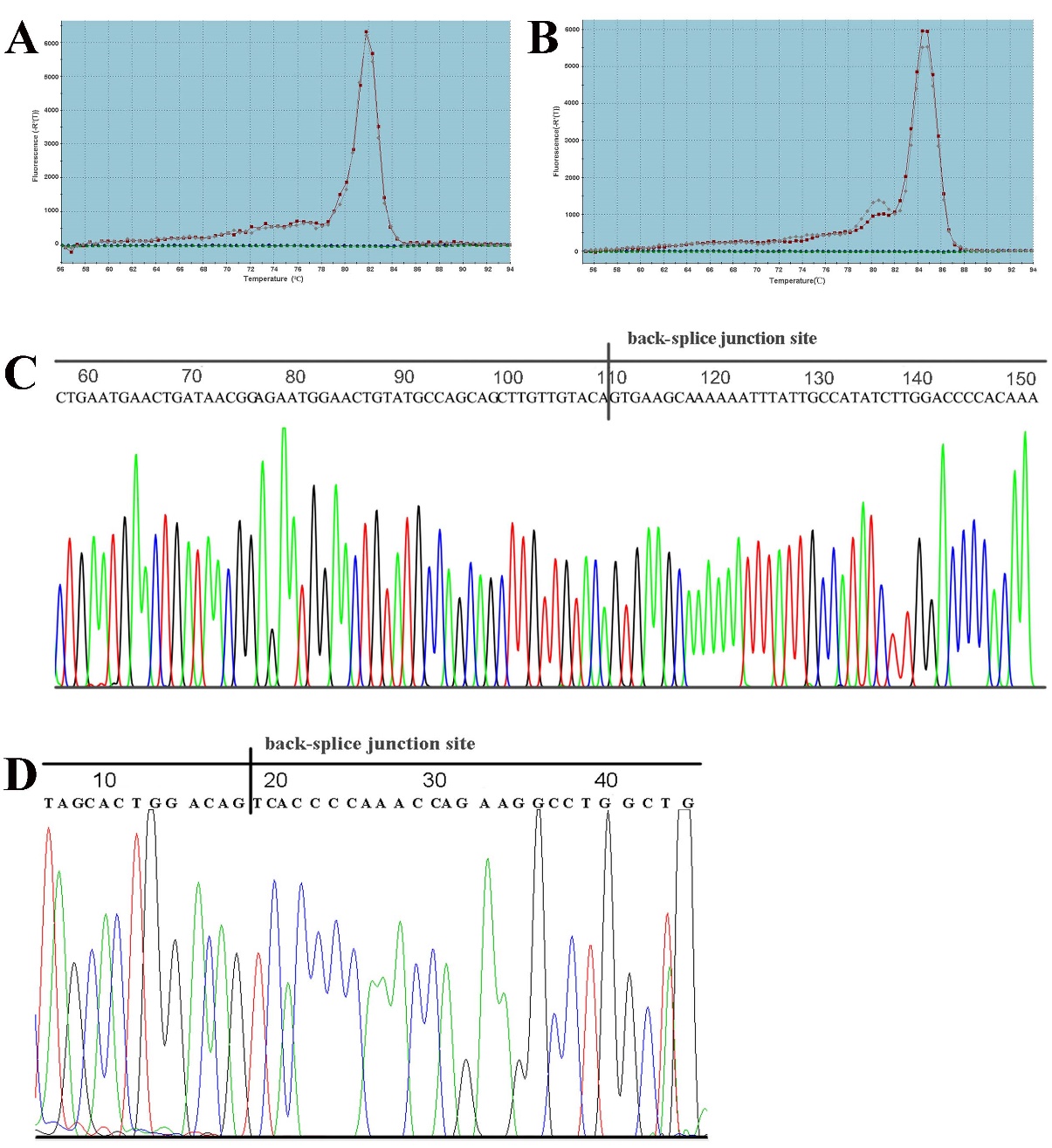


Figure S2. The immunohistochemical (IHC) images of tissue CEA (A, B) and CA19-9 (C, D).


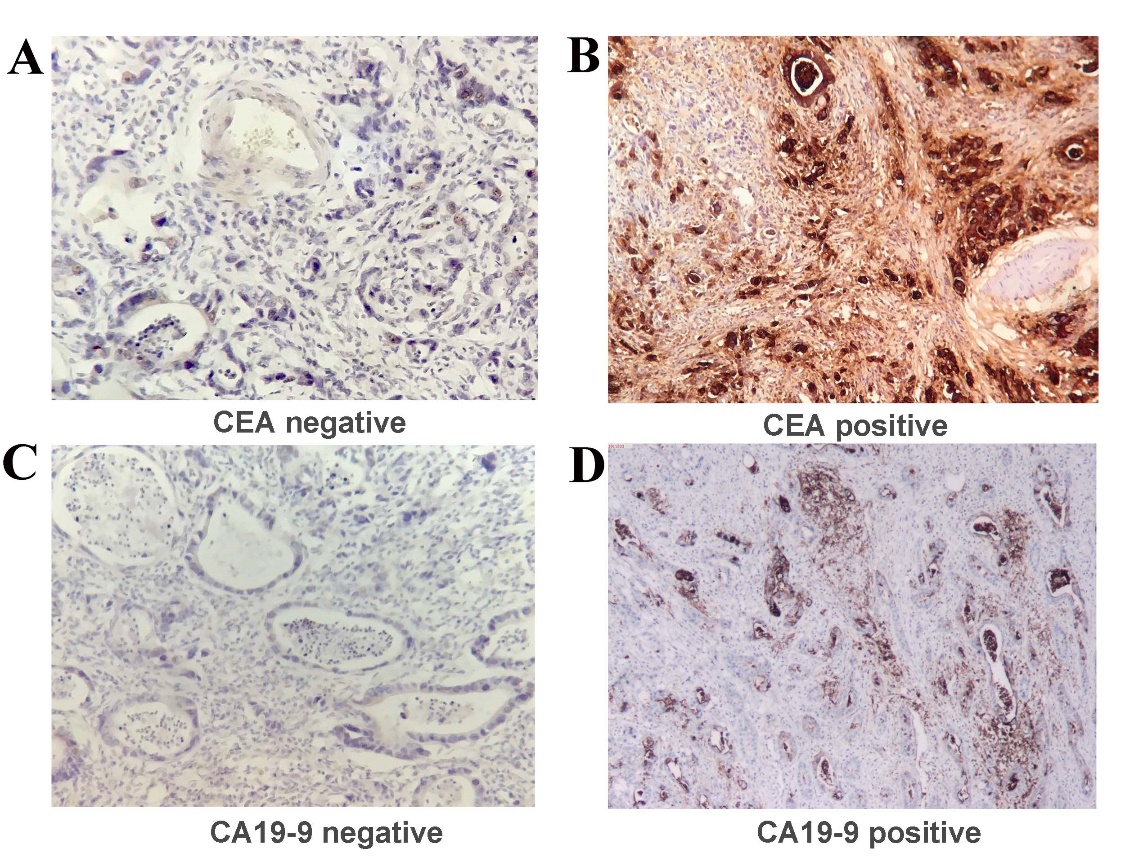

Supplement: Supplementary file 1 [file JCLA-33-na-s001.docx]
